# Progression to myocardial infarction short-term death based on interval sequential pattern mining

**DOI:** 10.1186/s12872-023-03393-7

**Published:** 2023-08-10

**Authors:** Yang-Sheng Wu, David Taniar, Kiki Adhinugraha, Chao-Hung Wang, Tun-Wen Pai

**Affiliations:** 1 Computer Science and Information Engineering, National Taipei University of Technology, Taipei, 106344 Taiwan; 2 Clayton Faculty of Information Technology, Monash University, Melbourne, VIC 3800 Australia; 3 Computer Science & Information Technology, La Trobe University, Melbourne, VIC 3086 Australia; 4 Heart Failure Research Center, Division of Cardiology, Department of Internal Medicine, Chang Gung Memorial Hospital, Keelung, 204201 Taiwan; 5 Chang Gung University College of Medicine, Taoyuan, 333010 Taiwan

**Keywords:** myocardial infarction, Short-term death, Disease trajectory, Interval sequential pattern mining

## Abstract

**Background:**

Myocardial infarction (MI) is one of the significant cardiovascular diseases (CVDs). According to Taiwanese health record analysis, the hazard rate reaches a peak in the initial year after diagnosis of MI, drops to a relatively low value, and maintains stable for the following years. Therefore, identifying suspicious comorbidity patterns of short-term death before the diagnosis may help achieve prolonged survival for MI patients.

**Methods:**

Interval sequential pattern mining was applied with odds ratio to the hospitalization records from the Taiwan National Health Insurance Research Database to evaluate the disease progression and identify potential subjects at the earliest possible stage.

**Results:**

Our analysis resulted in five disease pathways, including “diabetes mellitus,” “other disorders of the urethra and urinary tract,” “essential hypertension,” “hypertensive heart disease,” and “other forms of chronic ischemic heart disease” that led to short-term death after MI diagnosis, and these pathways covered half of the cohort.

**Conclusion:**

We explored the possibility of establishing trajectory patterns to identify the high-risk population of early mortality after MI.

## Background

According to a report from the Taiwan Ministry of Health and Welfare, cardiovascular diseases (CVDs) were the second significant cause of death in 2021, surpassed only by the combination of all types of cancer, leading to 93.1 deaths per million people, increasing for two consecutive years from 84.2 in 2019 [1]. Among these, myocardial infarction (MI) is one of the significant CVD diseases [2]. Although the incidence rate remained constant in recent years, the age of occurrence tended to be younger [3]. Besides, it is necessary to pay more attention to the substantial risk rising of MI in warm-climate cities including Taiwan [4].

MI, which is necrosis of the heart muscle caused primarily by a decrease in or stoppage of blood flow to a portion of the heart, is a severe CVD [5]. In the past 25 years, billions of dollars have been spent on new therapies, resulting in a slight reduction in adverse events, but a significant residual event rate remains [6]. Strategies aimed at preventing MI may be better for improving patient outcomes. We should focus efforts on the asymptomatic stage before symptoms occur due to the onset of atherosclerosis. Therefore, earlier identification and treatment are required to achieve a lower event rate [7, 8].

**Fig. 1:**
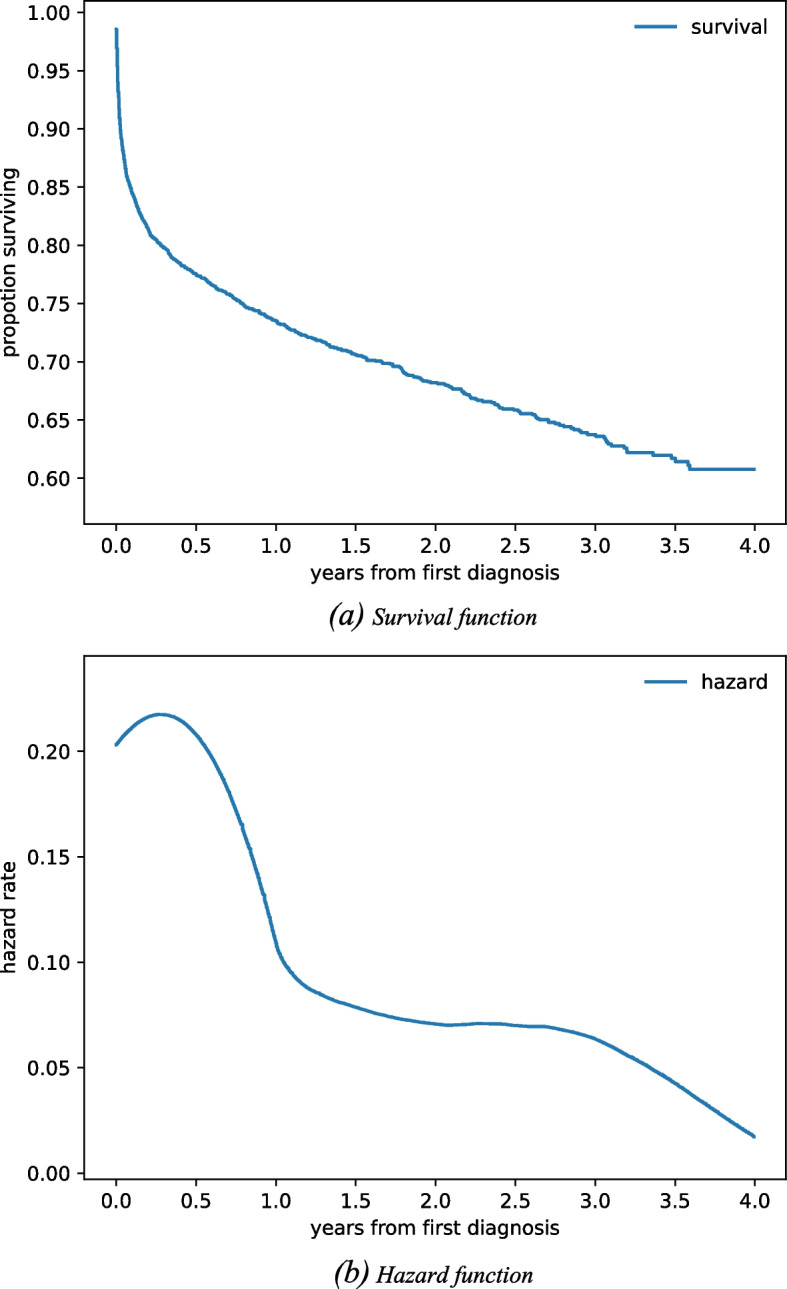
Survival functions of myocardial infarction patients in Taiwan from 2010 to 2013 after the first diagnosis. Both functions share a horizontal timeline, originating from year zero when the patient was first diagnosed

The first year after MI is noted as a particularly vulnerable period [9, 10]. According to our Taiwan health insurance database analysis, the hazard rate reaches a peak in the first year after the diagnosis, then drops to a relatively low value and maintains stable for the following years (Fig. 1.(b)). In other words, specifying the associated risk factors of early death after MI diagnosis may assist patients in pursuing prolonged survival [11]. Consequently, our objective was to identify suspicious comorbidities before diagnosis that may bring out short-term death.

In the present study, we applied an interval sequential pattern mining algorithm to Taiwan health insurance data to uncover disease trajectories showing the progression before MI occurrence in at-risk patients. We believe this will help to understand the development of MI and the risks associated with short-term death and to identify potential patients earlier. Compared to the state-of-the-art method, which started with identifying disease pairs and directions and then combining overlaps into disease trajectories [12, 13], another partitioning-based method, by clustering individuals sharing the same behavior as trajectories [14], we adopted a pattern mining technique for new possibilities. Our proposed approach aims to locate intact pathways founded on the individuals, including crucial temporal information and the intervals between admissions, enabling the potential of analyzing re-hospitalizations and corresponding interval changes between hospital stays.

## Methods

### Taiwan myocardial infarction data

The data source was the Taiwanese National Health Insurance Database (NHIRD) [15], including two datasets: the patient demographic dataset selected from Taiwanese people diagnosed with MI from 2010 to 2013; and the hospitalization dataset, including corresponding inpatient records spanning 1996 through 2013. Both datasets are de-identified to meet the obligations under the privacy act.

The hospitalization dataset contained 18,875 inpatient records including information on the date of diagnosis and at least one to five diagnoses encoded using ICD-9-CM. ICD-9-CM, “International Classification of Diseases, Clinical Modification,” is a hierarchical classification system for assigning diagnostic and procedure codes [16]. To represent a diagnosis, the format of the code is three digits that refer to an individual disease and two digits after the decimal point that refer to the detail of the disease. Since we only focused on individual diseases, we discarded the two digits after the decimal point, resulting in three-digit ICD-9 codes denoting disease diagnoses. Another patient demographic dataset selected subjects from the 1-million population dataset and included information on gender, birth date, and death date (the death date was None if patients were still alive at the final collection date of 2013–12-31).

### Study design

**Fig. 2:**
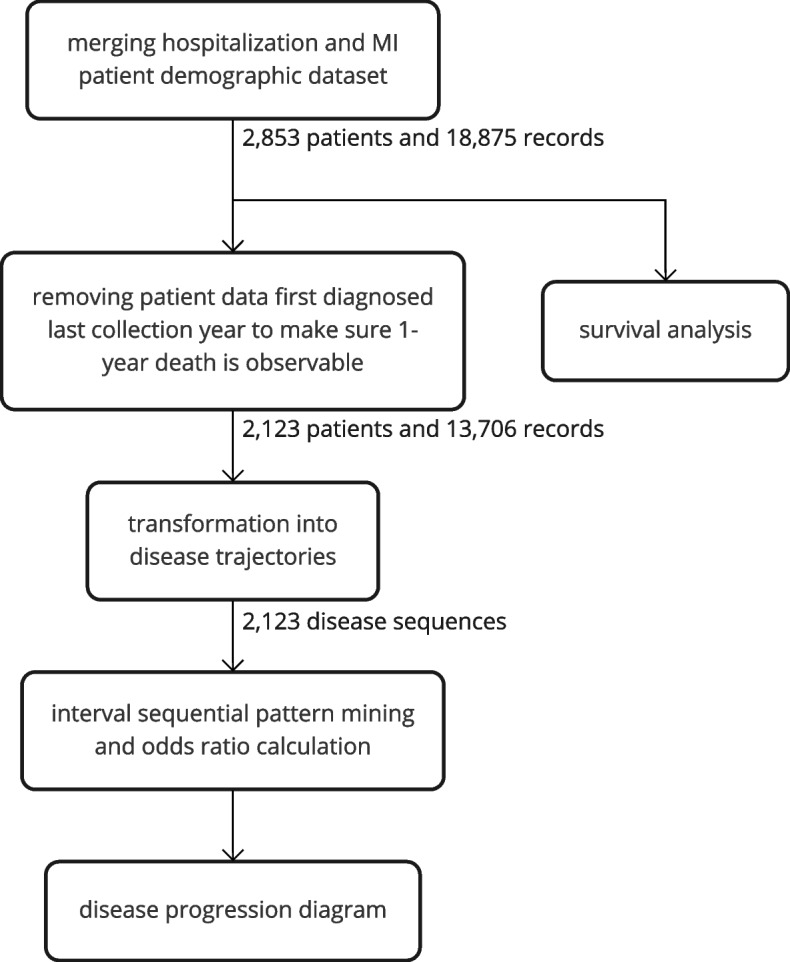
Flowchart of the study. The data contains two datasets from Taiwan’s health insurance database, including the patient demographic dataset and the hospitalization records

Figure 2 illustrates the stages of the study. The initial stage involved merging of the two datasets and retrieval of the corresponding hospitalization records. We first performed a survival analysis for the patients and defined death within 1 year as short-term death. Afterward, to prepare for the subsequent odds ratio calculation, we removed subjects diagnosed in the last data collection year, 2013, to ensure that short-term death was observable for all subjects. A total of 730 subjects were removed, and 2,123 subjects with 13,707 related inpatient records remained. We then transformed these data into trajectories involving the diagnoses and the date for the subsequent mining process. In the final stage, we applied interval sequential pattern mining and filtered significant patterns based on the odds ratio. While combining these patterns, we established a disease trajectories diagram to demonstrate the disease progression.

### Merging hospitalization and patient demographic dataset

This study comprised a patient demographic dataset and a hospitalization dataset. We merged the two datasets and retrieved the first diagnosis date for each patient. The first diagnosis date of our target disease, “myocardial infarction,” was defined as the date the patient was first hospitalized with the disease. There were 2,853 patients plus 18,875 hospitalization records covering the hospitalization history of patients from 1996 to the first diagnosis date.

### Survival analysis and 1-year short-term death

Survival analyses contain tools to estimate the interval from an event to death for a group of people. There are some typical problems associated with analyzing survival datasets. One is censoring. Generally, a study has a data collection range, but not all the events of interest occur before the end of the collection time, and therefore, right censoring is necessary. For instance, in our dataset, the last collection date was 2013–12-31, and the event we were interested in was death. However, not all the patients passed away before this date, so we could not observe the mortality of all individuals. However, discarding these individuals would waste knowledge and cause bias. Another problem is the skewed distribution of survival data, which fails most statistics tools based on Gaussian distribution. A popular way to solve this problem is to use the Kaplan–Meier estimator. We could adopt censored data to construct the survival function with a step function. Meanwhile, we were curious about the hazard value at each given time. Unfortunately, the Kaplan–Meier estimator does not work well in constructing the hazard function. Another method is called the Nelson–Aalen estimate [17, 18]. We applied both estimators to our dataset for survival and hazard values from the first MI diagnosis to death [19].

From observation in Fig. 1, we found a significant decrease in survival rates at the beginning of the curve, indicating that many people die right after the first diagnosis. When we arrived at the hazard function, with a 1-year bandwidth smoother, the result demonstrated a high risk of passing away after the diagnosis. After this 1-year peak, the hazard becomes lower and stable. Accordingly, we decided to base our investigation around this 1-year peak.

### Transformation into disease trajectories

**Fig. 3:**
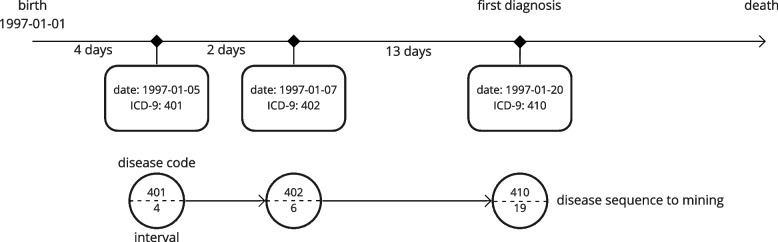
Example of transformation of hospitalization records into disease trajectory with intervals. The top line represents a person’s timeline; for example, birth 1997–01-01 and first diagnosis 1997–01-20. This timeline contains some records of visits with the hospitalization date and diagnoses. Information on diagnoses is obtained and transformed into a disease sequence with the intervals noted from birth

Before applying the mining algorithm, we removed subjects who were diagnosed in the last year of our final data collection year (2013) to guarantee the observability of short-term death. We then labeled each patient based on whether he or she passed away within 1 year after the first diagnosis. Of 2,123 patients, 592 (27.89%) passed away in the short-term, and 1,531 (72.11%) did not. Next, hospitalization records for each patient were extracted and transformed into sequences. Sequences included all records until the date of the first diagnosis of MI (Fig. 3).

### Interval sequential pattern mining and odds ratio calculation

Yu Hirate and Hayato Yamana proposed an algorithm in 2006 for generalizing interval sequential pattern mining—constraint-based and extended sequence-based mining. The algorithm handles seven inputs: interval-extended sequence database, interval itemization function to convert item intervals to pseudo items, minimal support of the pattern, and four types of constraints—min/max interval between items and min/max interval of the whole sequence. This function lets $I=\{{i}_{1}, {i}_{2}, \dots ,{i}_{n}\}$ be a set of all items, and X is a subset of items sorted alphabetically. A sequence is denoted by $\langle \left({t}_{\mathrm{1,1}},{X}_{1}\right),\left({t}_{\mathrm{1,2}},{X}_{2}\right),\dots ,\left({t}_{1,n},{X}_{n}\right)\rangle$, where${t}_{\alpha ,\beta }={X}_{\alpha }.time-{X}_{\beta }.time$, and${X}_{\alpha }.time$, ${X}_{\beta }.time$ represent the transaction occurrence time. The output is a sequential pattern with intervals satisfying the minimal support and constraints. A pattern is denoted by $<\left(\Delta {t}_{1},{x}_{1}\right),\left(\Delta {t}_{2},{x}_{2}\right),...,\left(\Delta {t}_{n},{x}_{n}\right)>$ where $\Delta t$ is the itemized interval and $x\in I$ [20]. With this algorithm, we fed our disease trajectories into the logarithm function (Eq. 1, Table 1), which we found worked better in convergent patterns after some trials, and we set a minimal support of 100 to obtain patterns.

**Table 1:** Lookup table for the logarithm function from item interval to pseudo item

Item interval	Pseudo item
0 days–7 days	0
7 days–21 days	1
21 days–35 days	2
1.17 months–1.63 months	3
1.63 months–3.50 months	4
3.50 months–7.23 months	5
7.23 months–14.70 months	6
1.23 years–2.47 years	7
2.47 years–4.95 years	8
4.95 years–9.94 years	9

Logarithm itemization function

1 $$I\left(t\right)=\lfloor{\mathrm{log}}_{2}\left(\frac{days}{7}+1\right)\rfloor$$

We then calculated the odds ratio by dividing patients into four groups—exposed/not exposed to patterns and short-term death/not short-term death—for each identified pattern to measure the strength of association. The odds ratio was used to indicate the effect size for categorical outcomes. An odds ratio of 1 meant the event was comparable in exposed and not exposed (control) groups. An odds ratio greater than 1 meant the event was associated with an increased risk in the exposed group. We set an odds ratio greater than 2, which is generally considered clinically significant [21], as a necessary condition to filter patterns.

## Results

### Interval sequential patterns

There were 14 interval sequential patterns identified. In Table 2, each row represents a pattern, including sequence, support (in total), support in not short-term death, support in short-term death, and odds ratio. The sequence was composed of a list of pairs: the first element of the pair was the pseudo item between the current and the previous pair, and the second element of the pair was the disease ICD-9 code format with three digits denoting a disease diagnosis. Support (in total), support in not short-term death, and support in short-term death included the count and occurrence rate among 2,123, 1,531, and 592 patients, respectively. The odds ratio was calculated based on exposure or no exposure to the pattern and whether death occurred within 1 year or not.

**Table 2:** Interval sequential patterns leading to myocardial infarction short-term death

Sequence	Support	Support in NSD	Support in SD	Odds ratio
< (0, 599), (7, 410) >	101 (4.76%)	38 (2.48%)	63 (10.64%)	4.68
< (0, 250), (7, 250), (8, 410) >	113 (5.32%)	51 (3.33%)	62 (10.47%)	3.39
< (0, 250), (9, 410) >	219 (10.32%)	115 (7.51%)	104 (17.57%)	2.62
< (0, 401), (9, 410) >	219 (10.32%)	118 (7.71%)	101 (17.06%)	2.46
< (0, 599), (8, 410) >	108 (5.09%)	57 (3.72%)	51 (8.61%)	2.44
< (0, 402), (9, 410) >	100 (4.71%)	53 (3.46%)	47 (7.94%)	2.4
< (0, 250), (8, 250), (7, 410) >	116 (5.46%)	62 (4.05%)	54 (9.12%)	2.38
< (0, 250), (6, 250), (8, 410) >	109 (5.13%)	59 (3.85%)	50 (8.45%)	2.3
< (0, 250), (7, 250), (7, 410) >	127 (5.98%)	69 (4.51%)	58 (9.80%)	2.3
< (0, 250), (6, 250), (7, 410) >	116 (5.46%)	63 (4.11%)	53 (8.95%)	2.29
< (0, 402), (7, 410) >	106 (4.99%)	58 (3.79%)	48 (8.11%)	2.24
< (0, 250), (7, 410) >	306 (14.41%)	176 (11.50%)	130 (21.96%)	2.17
< (0, 414), (8, 410) >	169 (7.96%)	97 (6.34%)	72 (12.16%)	2.05
< (0, 414), (9, 410) >	112 (5.28%)	64 (4.18%)	48 (8.11%)	2.02

The patterns are sorted by descending odds ratios. Each row represents a pattern with five columns: sequence, support (in total), support in not short-term death (NSD), support in short-term death (SD), and the odds ratio. A sequence is a list of pairs. The first element of the pair is the pseudo item between the current and the previous pair (the interval of the first pair is always zero), and the second element is the diagnosis encoded in ICD-9-CM codes^1^. The odds ratio is calculated against short-term death

^1^The corresponding diseases of ICD-9-CM codes are 250 for “diabetes mellitus,” 401 for “essential hypertension,” 402 for “hypertensive heart disease,” 410 for “myocardial infarction,” 414 for “other forms of chronic ischemic heart disease,” and 599 for “other disorders of the urethra and urinary tract,” respectively

We identified five groups from these patterns, including “diabetes mellitus” (250), “other disorders of the urethra and urinary tract” (599), “essential hypertension” (401), “hypertensive heart disease” (402), and “other forms of chronic ischemic heart disease” (414) based on initiating disease.

The highest support of the patterns was 306, accounting for 14.41% of the total population and 21.96% of those who died in the short-term. In other words, at least one of five patients who died in the short-term demonstrated the pattern. The pattern started with “diabetes mellitus” hospitalization, after 7-item intervals (1.23 to 2.47 years), then diagnosis with “myocardial infarction,” with a 2.17 odds ratio of short-term death. Compared to the patients not exposed, these patients had more than double the chance of passing away within 1 year after diagnosis. In addition, we found re-hospitalization for diabetes mellitus in similar expressions.

Furthermore, the highest odds ratio was 4.68 for these patterns. This pattern with a support of 101 started with “other disorders of the urethra and urinary tract” (599) after 7-item intervals, then diagnosis with “myocardial infarction.” Another pattern started with “other disorders of the urethra and urinary tract” after 8-item intervals (2.47 to 4.95 years), then moved to “myocardial infarction” with a support of 108 and a 2.44 odds ratio.

Next were “essential hypertension” and “hypertensive heart disease,” which shared the same ICD-9 category, “hypertensive disease” (401–405). The pattern of “essential hypertension” was identified, through 9-item intervals (4.95 to 9.94 years) and arrival at “myocardial infarction.” Another two patterns from “hypertensive heart disease” were identified. One was 7-item intervals before “myocardial infarction” and another was 9-item intervals before “myocardial infarction.”

We discovered that “other forms of chronic ischemic heart disease” disease belonged to the ICD-9 category “ischemic heart disease,” which is the same as “myocardial infarction.” We identified two similar patterns for the disease. The intervals between “other forms of chronic ischemic heart disease” to “myocardial infarction” were 8-item intervals with a support of 169 and a 2.05 odds ratio, and another was 9-item intervals with a support of 112 and 2.02 odds ratio to short-term death.

### Disease trajectory diagram

There were five groups of patterns. We combined these patterns and item intervals into five trajectories to MI short-term death.

The first was “diabetes mellitus.” We found that re-hospitalization for this disease was associated with “myocardial infarction” short-term death. Combining the adjacent node to “myocardial infarction,” the first edge was constructed with item intervals from 7 to 9 (1.23 to 9.94 years) and an odds ratio from 2.17 to 2.62. Then, we made a new edge of the previous node with item intervals from 6 to 7 (7.23 months to 4.95 years) and an odds ratio from 2.29 to 3.39.

**Fig. 4:**
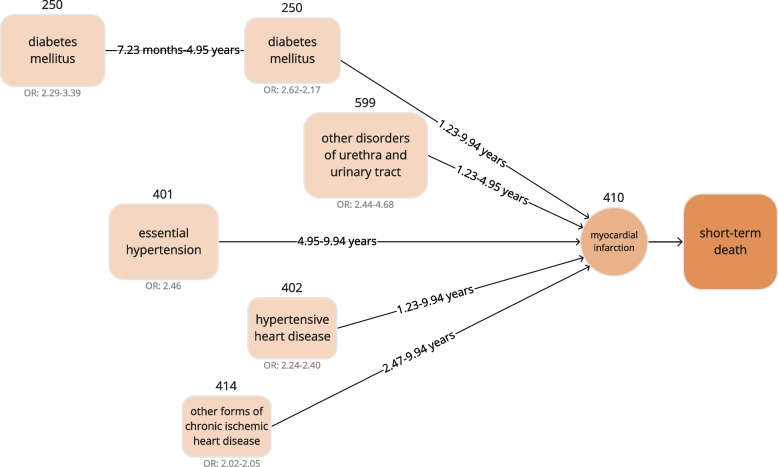
The disease trajectory diagram before the first diagnosis showing significant pathways direct to short-term myocardial infarction death (OR, odds ratio)

The same procedure was applied for the rest of the sequences. By merging the intervals and odds ratios, we completed four disease trajectories. Based on the last node, “myocardial infarction,” the disease trajectory diagram of the progression of “myocardial infarction” short-term death was established by combining all the trajectories—five trajectories with intervals covering more than half (52.20%) of the short-term death population. Among these, short-term death patients had an average of 2.18 patterns in the trajectories compared to 1.08 patterns for not short-term death patients. We observed a more considerable difference in the median value of 2 for short-term death compared to 0 for not short-term death (Fig. 4).

## Discussion

### Medical mechanism

In this study, we used an interval sequential pattern mining algorithm approach to gain knowledge from our data source. An association between all identified patterns and mortality after MI was frequently identified in our dataset. However, some patterns were not intuitive in terms of understanding the associated mechanisms. For example, one of the trajectories in the diagram was related to urinary tract infections (UTIs). Clinically, recurrent UTIs may indicate the presence of inflammation or impaired immune function. Usually, chronic inflammation is associated with the development of CVD and may also make stable vascular plaque vulnerable and unstable. In response to active inflammation, these plaques are subject to “rupture” to cause MI. At the same time, inflammation may cause increased oxidative stress, which is correlated with cardiovascular attacks and the worsening of other systemic diseases. Furthermore, UTI is one of the complications related to systemic diseases such as diabetes mellitus and stroke. Hence, a history of UTI may suggest the existence of these systemic diseases with poor outcomes once MI occurs. These potential mechanisms may explain why the risk of short-term mortality increased 4.68-fold when a patient had MI preceded by a trajectory related to UTI.

On the other hand, we found a trajectory of re-hospitalization for diabetes mellitus in another pattern. Common causes of hospitalization for diabetes mellitus include poor sugar control or infection. Poor sugar control is directly related to several complex situations. First, poor diabetes mellitus control is closely related to diffuse atherosclerosis in the cardiovascular system. Once a trajectory of recurrent hospitalization due to poor sugar control is noted, the occurrence of MI is usually associated with multi-vessel coronary artery disease. This multi-vessel disease is complicated and often is correlated with higher mortality. Second, poor sugar control is associated with accelerated atherosclerosis. Accelerated atherosclerosis after MI may cause a heart attack in the near future. Third, cardiomyopathy is quite often noted in patients with poor diabetes mellitus control. MI, along with either asymptomatic or symptomatic heart failure, may definitely result in poor outcomes, such as early mortality. Fourth, patients with poor diabetes mellitus control usually have multiple comorbidities, such as kidney disease, cerebral vascular disease, and peripheral artery occlusive disease. Multiple comorbidities substantially increase the mortality risk after MI. Finally, these patients with poor diabetes mellitus control are subject to infection, which is one of the well-known risk factors for mortality after MI.

### Limitations

We applied interval sequential pattern mining on the EMR dataset to interpret different disease trajectories and show the progression to short-term MI death. Despite this, some limitations exist compared to the established bottom-up approach published in Nature Communication 2014 [12]. Because of the nature of the algorithm, this approach performs poorly in terms of constructing a long trajectory. Moreover, we used the interval itemization function to differentiate the patterns but choosing a proper itemization function is a problem. In our study, we used a weekly logarithm itemization function and discovered some interesting patterns. However, it is not possible to develop an optimized itemization function. Different function settings may result in different patterns in diverse views.

## Conclusion

This study explored the possibility of applying an interval sequential pattern mining algorithm to Taiwan EMR data to generate disease trajectories.

Using the approach adopted in our study, we established a few patterns of trajectory to identify the high-risk population for early mortality after MI. Our algorithm needs further validation in an independent population and also warrants clinical trials to see if more aggressive interventions can lower the mortality rate in this population. We hope our findings will provide additional knowledge about the factors that lead to short-term death and assist early identification of potential at-risk patients.

## Data Availability

The patient demographic and hospitalization datasets are from the NHIRD and were used only for this study under policies limitation. Therefore the data is not publicly available. Interested researchers can obtain the data through the formal application (https://nhird.nhri.org.tw/en/).
